# When MALDI-TOF MS and 16S rRNA gene sequencing are not enough: identification of *Gordonia hongkongensis* causing bloodstream infection

**DOI:** 10.1128/asmcr.00007-26

**Published:** 2026-04-24

**Authors:** Ariel Gianecini, Maria Florencia Abeiro, Florencia Rocca, Sergio Daniele, Juan Francisco Losinno, Lucia Cipolla, Maria Virginia Gonzalez

**Affiliations:** 1 Special Bacteriology Service, National Institute of Infectious Diseases—ANLIS “Dr. Carlos G. Malbrán”, Ciudad Autónoma de Buenos Aires, Argentina; 2 Hospital Italiano de la Plata, Provincia de Buenos Aires, Argentina; Rush University Medical Center, Chicago, Illinois, USA

**Keywords:** *Gordonia hongkongensis*, bloodstream infection, immunocompromised host, whole-genome sequencing

## Abstract

**Background:**

*Gordonia* spp. are gram-positive aerobic bacilli whose infections in humans are rare and typically affect immunocompromised hosts and those with indwelling medical devices. Conventional identification systems frequently misidentify *Gordonia* species. Here, we present a bloodstream infection caused by *Gordonia hongkongensis*.

**Case Summary:**

A 76-year-old man with metastatic colorectal adenocarcinoma receiving oral chemotherapy presented with febrile syndrome and acute diarrhea, unresponsive to prior antimicrobial therapy. Blood cultures were positive for gram-positive rods, which grew producing pink-to-orange pigmented colonies. Matrix-assisted laser desorption ionization–time of flight mass spectrometry (MALDI-TOF MS) and 16S rRNA gene sequencing could not distinguish the isolate at the species level, identifying it as belonging to the genus *Gordonia*. Owing to the limitations of these methods, whole-genome sequencing (WGS) was performed, which revealed *G. hongkongensis* as the causative pathogen. Updating the MALDI-TOF MS database with the main spectral profiles enabled the correct identification of the isolate upon reanalysis. Empirical antimicrobial therapy with piperacillin–tazobactam was initiated before organism identification and susceptibility testing. After a 7-day course of treatment, the patient showed clinical improvement and resolution of fever.

**Conclusion:**

Human infections with *G. hongkongensis* are rare. This case underscores the diagnostic challenges associated with *Gordonia* species and emphasizes the role of WGS in accurately identifying emerging pathogens. Increased awareness and improved diagnostic tools are essential for a better understanding and management of *Gordonia* infections.

## INTRODUCTION

*Gordonia* spp. are gram-positive, rod-shaped, aerobic actinomycetes that are widely distributed throughout natural environments and frequently found in soil and water (1). Although human infections are rare, systemic bloodstream infections, postoperative or traumatic infections have been reported, mainly in patients with underlying immunodeficiencies or contaminated endovascular devices (2, 3).

More than 50 *Gordonia* species have been described (data from LPSN, https://lpsn.dsmz.de/genus/gordonia); however, only a few have been associated with human diseases (1). Identification at the genus and species level by conventional microbiology culture and biochemical analysis is difficult and sometimes leads to misidentification as *Corynebacterium*, *Nocardia,* and *Rhodococcus* species (4). The use of 16S rRNA gene sequencing and matrix-assisted laser desorption ionization–time of flight mass spectrometry (MALDI-TOF MS) systems has improved *Gordonia* identification rate (5). Nevertheless, in some cases, whole-genome sequencing (WGS) is required to achieve definitive taxonomic classification (6). These limitations may contribute to the underestimation of the diversity of *Gordonia* species associated with human infections.

Here, we present a case of *Gordonia hongkongensis* bloodstream infection in an immunocompromised patient, identified using WGS. To our knowledge, this is the second report of *G. hongkongensis* infection to date, contributing to the expanding knowledge of the clinical spectrum, management approaches, and outcome of this uncommon species.

## CASE PRESENTATION

A 76-year-old male with a history of colorectal adenocarcinoma with liver metastases, currently receiving oral chemotherapy (trifluridine/tipiracil, bevacizumab, and filgrastim), presented to the emergency service with a febrile syndrome and acute diarrhea of 48 h duration. He reported a 20-day course of amoxicillin–clavulanate therapy prescribed for non-severe pneumonia prior to presentation, without clinical improvement. On admission, body temperature was 38°C, oxygen saturation was 99% (reference range: 95%–100%), peripheral white blood cell counts of 5,340/mm^3^ (reference range: 4,000–10,000/mm^3^), and 50% neutrophils (reference range: 42%–72%). The chest radiography showed diffuse interstitial infiltrates involving both lung bases. The initial infectious diseases evaluation included a viral Panbio COVID-19/Flu A&B panel (Abbott GmbH & Co. KG, Wiesbaden, Germany), *Clostridioides difficile* GDH and toxin A/B (Hangzhou AllTest Biotech Co., Ltd.), as well as one set of two aerobic blood cultures (BACTEC FX; Becton Dickinson, Cockeysville, USA). Results of the viral panel and toxin assay were negative. He was started on prophylactic antimicrobial treatment with intravenous piperacillin–tazobactam (PTZ) at a dose of 4.0/0.5 g every 4 h, infused over 30 min.

**Fig 1:**
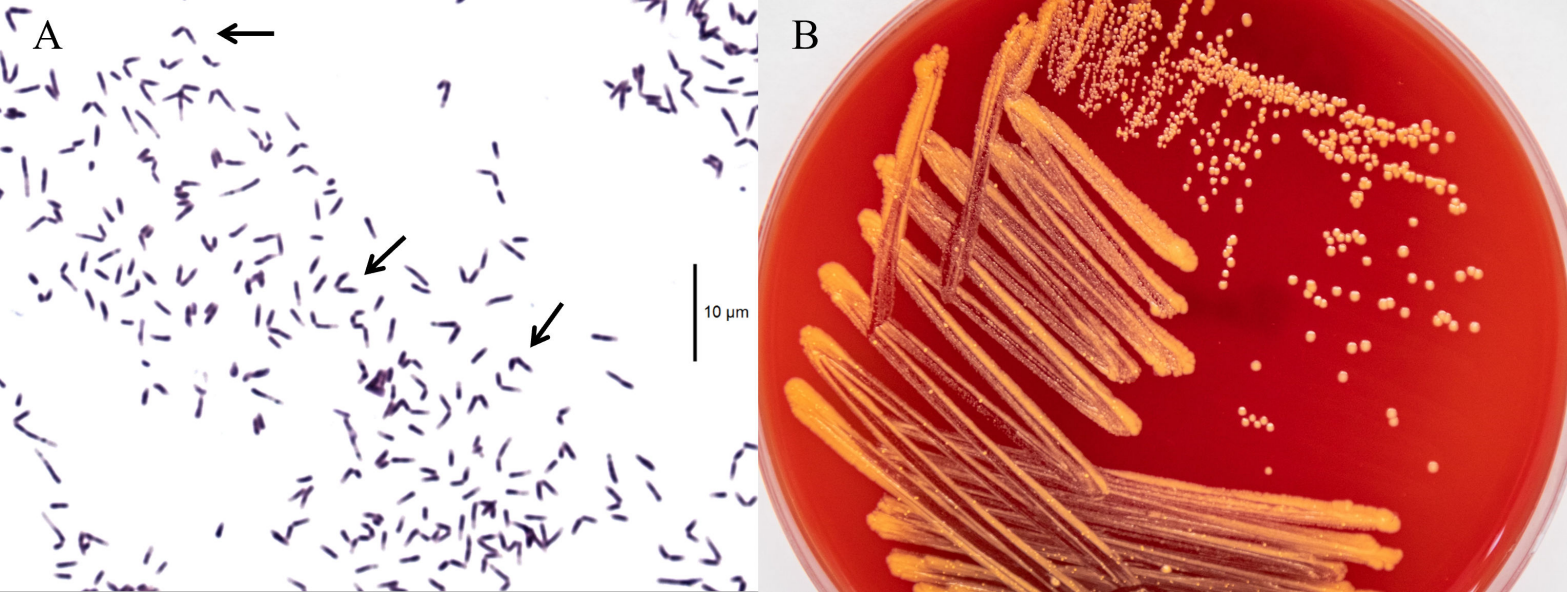
(**A**) Gram stain showing diphtheroid gram-positive rods from the colony after 24 h incubation. The arrows indicate the characteristic V-shaped arrangement of the bacterial cells. (**B**) Orange colonies on the 5% sheep blood agar medium after 3 days of incubation.

**Fig 2:**
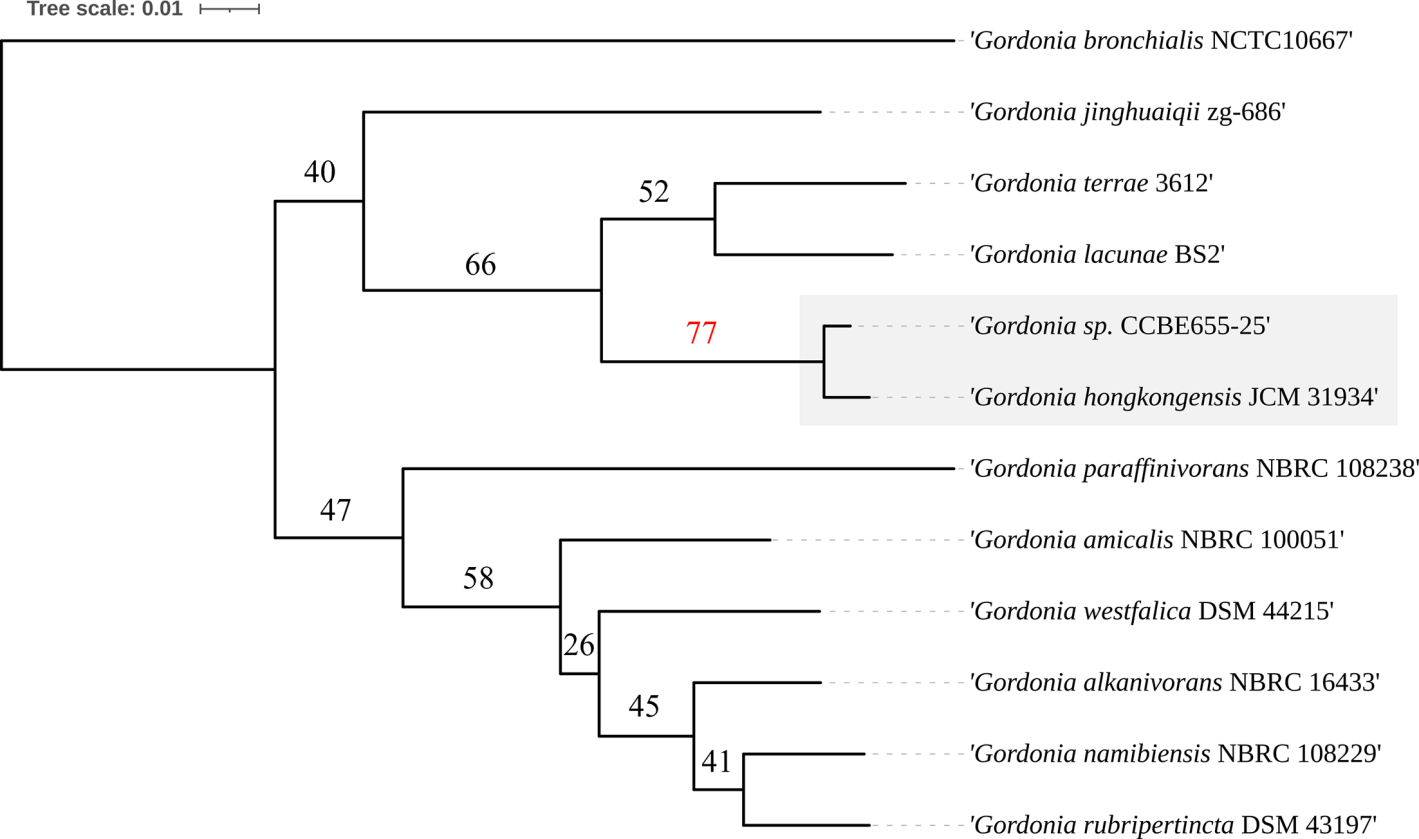
Phylogenetic tree constructed using core genomic data of *Gordonia* species and *Gordonia* sp. strain CCBE655-25. All closely related *Gordonia* species identified by TYGS and GTDB-tk were included. Genome sequences of type strains of *Gordonia* species were obtained from NCBI (https://www.ncbi.nlm.nih.gov/). A gray box highlights the position of the *G. hongkongensis* reference strain (JCM 31934) and *Gordonia* sp. strain CCBE655-25, and the branch support value is shown in red.

On day 2 of hospitalization, blood cultures collected on admission were positive with growth in two of the two bottles collected within 48–72 h. The Gram stain showed gram-positive rods. The organism grew on 5% sheep blood agar and chocolate agar within 24 h of incubation at 37°C and 5% CO_2_ as small, pink-to-orange, non-hemolytic colonies and was confirmed as gram-positive (Fig. 1). The isolate was referred to the National Reference Laboratory for genus and species confirmation. MALDI-TOF MS identification was performed using multiple commercial platforms, MALDI Biotyper Sirius (Bruker Daltonik, Bremen, Germany), VITEK MS PRIME (bioMérieux, France), and Autof MS 2600 (Autobio Diagnostics, Zhengzhou, China). All three systems identified the isolate (named CCBE655-25) as a different *Gordonia* species (Table 1). To confirm the taxonomic identification, the bacterial genome was subjected to WGS using the Illumina MiSeq platform (Illumina, CA, USA) and a 2 × 150 bp MiSeq Reagent Kit v2. Quality of raw data was assessed with FastQC v0.12.1 (https://www.bioinformatics.babraham.ac.uk/projects/fastqc/), and *de novo* assembly was performed using Unicycler v0.5.1 (7). The quality of the final assembly was verified using QUAST v5.1.0rc1 and annotated with Prokka v1.14.6 (8, 9). The full-length 16S rRNA gene (1,521 bp) of CCBE655-25 could not distinguish the isolate at the species level, showing 99.93% sequence identity with *Gordonia terrae* and *G. hongkongensis*, respectively (https://www.ezbiocloud.net/resources/16s_download) (10). The sequence of the 16S rRNA gene was further confirmed by Sanger sequencing. For taxonomic analysis, the *de novo* assembly was submitted to the Type (Strain) Genome Server (TYGS) to identify the nearest phylogenetic neighbors and calculate digital DNA-DNA hybridization (dDDH) values (https://tygs.dsmz.de/). The average nucleotide identity based on BLAST (ANIb) was performed on the JSpecies website (https://jspecies.ribohost.com/jspeciesws/). The assembly was additionally classified within the Genome Taxonomy Database using GTDB-Tk v2.4.1 (11). The phylogenomic position of the isolate was verified using the Up-to-date Bacterial Core Gene pipeline (v3.0) (12). Based on the 70% dDDH (88.7%) and 95%–96% ANIb (97.3%) thresholds, together with the GTDB classification, the isolate was identified as *G. hongkongensis* (13). Phylogenomic analysis further confirmed its taxonomic position, with 77 out of 81 core genes supporting this classification (Fig. 2). To strengthen MALDI-TOF identification of locally relevant and emerging pathogens, main spectrum profiles (MSPs) were generated using the MALDI Biotyper Sirius, according to the standard MALDI Biotyper MSP Creation method in the FlexControl 3.4 software (Bruker Daltonics), as previously described (14). The MALDI Biotyper Sirius platform enabled user-expandable MSP libraries through well-documented workflows, facilitating the database enhancement approach used in this study. MSPs were added to the in-house database, and reanalysis showed the best match with *G. hongkongensis* CCBE655-25 MSP (score > 2.0).

**TABLE 1:** CCBE655-25 isolate identification by three different MALDI-TOF MS platforms^*a*^

Platform	Database	Identification	Score/confidence (best match)
Sirius-Biotyper	v13.0.0.0	*Gordonia hongkongensis*	2.41
		*Gordonia terrae*	1.98
Vitek MS Prime	v3.3	*Gordonia terrae*	99.9
Autof MS 2600	Autof	*Gordonia rubripertincta*	9.56
		*Gordonia rubripertincta*	9.40

^*a*^ As *Gordonia* species are not included in the *in vitro* diagnostic databases of any of the three platforms, the CCBE655-25 isolate was analyzed using research-use-only (RUO) databases. Hence, according to CLSI M58 guidelines, additional studies are required to confirm identification when using RUO databases (15).

Antibiotic MICs were estimated using gradient strips (bioMérieux, Marcy l'Etoile, France) on Mueller-Hinton agar with a 0.5 McFarland standard inoculum. After 48 h of incubation, the MICs were as follows: piperacillin–tazobactam (0.06 µg/mL), amoxicillin/clavulanate (0.125 µg/mL), ceftriaxone (0.125 µg/mL), imipenem (0.016 µg/mL), meropenem (0.06 µg/mL), ciprofloxacin (0.008 µg/mL), clarithromycin (0.03 µg/mL), linezolid (0.5 µg/mL), minocycline (0.125 µg/mL), trimethoprim-sulfamethoxazole (0.25 µg/mL), and vancomycin (0.5 µg/mL). The patient was discharged on the 8th day of hospitalization following a 7-day course of PTZ treatment, with improvement of clinical signs and resolution of fever. Ten days later, a follow-up computed tomography scan of the chest showed no abnormality.

## DISCUSSION

*G. hongkongensis* is a gram-positive, weakly acid-fast, non-sporulating rod (diphtheroids) belonging to the *Gordonia* genus (16). Tsang et al. initially characterized this species in 2016 following its recovery from clinical samples, specifically from blood culture and peritoneal dialysis effluent (16). After the original description, no additional human infections with *G. hongkongensis* have been described. *Gordonia* species are ubiquitous bacteria that cause opportunistic infections in both immunocompromised and immunocompetent hosts (1). The most common medical history in patients included congenital or acquired immunodeficiencies, active immunosuppressive chemotherapy, hematological malignancies, implanted catheters and medical devices, and hematopoietic stem cell or solid organ transplant recipients (2, 3). Reported clinical presentations vary from catheter-related bacteremia, endocarditis, pneumonia, brain abscess, and skin and soft tissue infections (2, 3). In the current case, our patient presented a bloodstream infection during chemotherapy treatment for a metastatic tumor, consistent with prior reports. Based on clinical, microbiological, and imaging findings, and in the absence of indwelling catheters, we hypothesize that the organism may have gained access to the bloodstream via the respiratory tract. This case underscores that in patients with signs of infection and underlying immune conditions, gram-positive rods found in blood cultures should not be routinely dismissed as contaminating diphtheroids.

16S rRNA sequencing and MALDI-TOF platforms are becoming commonplace for bacterial identification in clinical laboratories (5). However, these methodologies have shown limitations in the accurate identification of *Gordonia* species (6, 17). In the present case, three commercial MALDI-TOF databases and 16S gene analysis were unable to achieve species-level identification within the genus *Gordonia*. WGS analysis ultimately identified the isolate CCBE655-25 as *G. hongkongensis*. Nevertheless, WGS is not yet widely available in most clinical laboratories, which may contribute to the underdiagnosis of *Gordonia* species infections. After creating MSPs, MALDI-TOF reidentification of the isolate with the updated in-house database yielded a confident species-level identification (score ≥ 2.0). This demonstrates that the identification of *Gordonia* species may be improved through the construction of a reference database with an increased number of spectra for each species. In medically critical conditions, isolates with discrepant or no initial identification should be referred to a reference laboratory for the proper identification of aerobic actinomycetes in order to further promote advancement in epidemiology, diagnosis, treatment, and clinical management of *Gordonia* infections.

There are no guidelines for the management of *Gordonia* infections, and reported cases have been managed heterogeneously. Previous reports have shown successful treatment using fluoroquinolones, vancomycin with or without aminoglycosides, and carbapenems, administered for 6–12 weeks (2, 3, 18). However, a recent report in patients with bacteremia showed that a short-term β-lactam treatment (1–2 weeks) may be considered with an aminoglycoside added in severe cases (19). Our patient was successfully treated using intravenous PTZ for 7 days, supporting the potential efficacy of this approach. Enhancing awareness of infections caused by *Gordonia* species and developing evidence-based treatment strategies could provide significant public health benefits.

In summary, we report a case of bloodstream infection caused by *G. hongkongensis*. Accurate species-level identification of this rare microorganism was challenging and required WGS, underscoring the importance of WGS for identifying emerging infectious agents. This report contributes to further understanding of the clinical significance and the challenges surrounding the identification of *Gordonia* species.

## Data Availability

The genomic assembly of CCBE655-25 was deposited in NCBI under BioProject accession no. PRJNA1394973.
